# The Bristol Medical Officer of Health

**Published:** 1922-09

**Authors:** 


### The Bristol Medico-Chirurgical Journal.

*" Scire est nescire, nisi id me*
*Scire alius sciret."*

SEPTEMBER, 1922.

### THE BRISTOL MEDICAL OFFICER OF HEALTH.

Every year for the past fifty years a volume of Reports dealing with the health conditions of our city has found its way into the library. We wonder how many have ever taken the trouble to look within the covers and learn how the immense population within the area comprised in our Urban Sanitary District has been protected from many a grievous outbreak of disease, how our good reputation as a city of health has been maintained, notwithstanding the special dangers that menace every great city which depends for much of its prosperity on its activities as a port. Yet the varied contents surely bear interest for most of us who follow the art of healing.

We doubt whether it is generally realised that throughout this long period the responsible chiefs of the department in our city have been firstly Dr. David Davies, Medical Officer of Health until 1886, thence onwards our present Medical Officer of Health, who succeeded his father.

The department now in charge of Dr. D. S. Davies is the latter-day development out of small beginnings when early in the nineteenth century there arose a widespread movement for the betterment of the industrial classes, more especially in regard to sanitation and all that tends to lessen disease.

The English epidemics of cholera in the years 1831, 1849 and 1854 directed attention to the urgent need for reform, particularly in ports like Bristol, a city that suffered so heavily from these outbreaks. Now cholera is one of the small group of diseases directly associated with primary sanitary defects, and this fact has tinctured all subsequent ideas in regard to the origin and spread of the communicable diseases generally, most of which, despite popular opinion, are not "merely a question of the drains."

Naturally the attention of the early reformers was directed to secure the three primary necessities for decent communal existence—pure air, pure water, pure soil; and in aiming at this, they were doubtless influenced by the then prevailing belief that "sanitation" was the panacea for all ills, and that all the communicable group of diseases had their origin in filth and defective drainage.

We gather from the Reports before us that it was a sharp visitation of typhus fever in 1865, followed by another introduction of cholera in 1866, which impelled the city to take advantage of permissive powers and to appoint their first Medical Officer (Dr. David Davies) to advise and initiate special measures for the control of disease. At that time also one of Bristol's most distinguished citizens, Dr. William Budd, was devoting his attention to the causation of disease, and with marvellous precision formulated views, far in advance of his time, which were accepted and acted upon with signal success by the Medical Officer. But it was not till late in the century that an attempt was made to provide Isolation Hospitals distinct from those provided by the Guardians. These, however, were at length established at Novers Hill and Ham Green, capable of extension as required for a growing city.

In addition to the routine treatment of the common communicable diseases by removal to hospital, disinfection and suchlike measures, we note that opportunity has been found for work of investigation or research into the natural history of disease, as evidenced in the Reports before us in regard to milk outbreaks of typhoid fever and of scarlet fever. We note with legitimate pride the pioneer investigations made in Bristol on the spread of certain diseases, *e.g.* diphtheria and typhoid fever by "carriers," that is by individuals who, although they themselves may appear in good health, nevertheless harbour the germs of the disease, which thereby spreads to others.

The responsibility for pulmonary consumption was at first left in our city to voluntary effort, and both Winsley Sanatorium and the first Tuberculosis Dispensary were provided in this way. They were subsequently taken over by the municipality with the central administration in the department of the Medical Officer of Health, in the scheme now comprising two dispensaries, in Portland Square and Redcliffe Parade, and beds for early and advanced cases at Ham Green as well as at Winsley. Quite recently the Frenchay Park scheme for dealing with its earliest manifestations in children has been developed.

A great impetus to the development of Infant Welfare and Maternity Work has necessitated a complete special department, now rivalling in size the whole previous Health Staff.

During the war a scheme was initiated for dealing with the prevalence of venereal disease, and in place of setting up costly *ad hoc* institutions, the Medical Officer of Health happily arranged with the Bristol Royal Infirmary and General Hospital to provide the statutory clinics for the treatment of venereal diseases.

It is deplorable, however, that the hospital accommodation for infectious cases has not kept pace with the growth of our population, and only last year the tuberculosis beds at the Ham Green Hospital had to be closed to make room for cases of diphtheria.

Some years ago, when we had a launch to take the Medical Officer out to visit suspected ships *before* entering our port, we were better equipped in certain respects for the checking of cholera and plague invasion than we are to-day, because once a ship with infectious disease on board has been docked the risks of introduction of disease are greater. The value of this launch was exemplified in 1894 when cholera was rampant in Hamburg, and existed in other Continental ports, for it was then possible to visit and inspect all incoming vessels from these ports before their entry, in accordance with the Ministry of Health's requirements. Then, again, in those days we possessed a shore port hospital and a hospital ship, whereas it appears that nowadays an infected ship can only be inspected after it is docked, and any serious infectious case is taken to Ham Green, where there is not even a separate building for such cases.

How will the city of Bristol keep its place in the van of progress without a more complete staff and equipment for port service ? We should take a leaf out of Liverpool's book, where these facilities which Bristol lacks are provided for the Medical Officer's department.

Our citizens sleep comfortably in their beds, accounting it a small thing that epidemics should be nipped in the bud. From time to time cases of *smallpox*, for instance, are introduced into the city, but since we have been fortunate enough to escape a serious outbreak of the disease we pay too little heed to the unseen and unheralded measures which have given us this immunity. We are convinced that the public at large does not recognise the unceasing vigilance and activity of our Medical Officer of Health and his staff, and the consequent saving in rates, as well as life and health.

Perchance more credit would be given if only, by the preventive measures being relaxed, a calamitous epidemic appeared in our midst to be stamped out with greater publicity and *éclat*—certainly more money would be spent. Let us turn to the Report of 1909 for one example in point. In 1908–9 three cases of smallpox were introduced, and the total outbreak resulting amounted to thirty-nine (15.4 per cent. hemorrhagic). We find that 16,000 separate inquiries were made by the Medical Officer of Health and staff, and the cost to Bristol was only £134 8s. 0d. To realise the saving in rates, compare with this the cost of a developed epidemic in 1901–2 in West Ham, of about the same population, which totalled £26,471 17s. 4d. !

Let us recall the threatened outbreak of a *plague* epidemic in August, 1916, when three cases, proved to be plague, occurred among workers at a St. Philip's rag warehouse, due to plague-infected rats. Most elaborate steps were immediately taken: the rats were exterminated, and every single individual that could possibly have become infected traced and kept under daily medical supervision, and an immense amount of labour expended in freeing houses of rats, or protecting them against invasion, etc. The report is most interesting reading. And the upshot ! The steps taken confined rat-plague to the warehouse ; had it spread to the outside rats the result would certainly have been disastrous to Bristol. We believe that a few years ago an outbreak in Glasgow originating under similar circumstances cost that city nearly one million pounds, apart from other more serious and irreparable losses.

Again, we may refer to the *typhoid* outbreak in 1897, when many cases developed in various houses in Clifton. Careful investigation showed that all these houses obtained their milk from a dairy at Bower Ashton. Here the source of the outbreak was located, and all the needful steps taken to stamp out further chances of mischief from this source. But only those who were working with our Medical Officer at that time can have any conception of the labour involved in thus tracking all the links in a chain of evidence which led to such a triumph in preventive medicine. Incidentally we note that this was the first typhoid epidemic in England in which the Widal test was systematically used in tracing cases.

Finally, as regards diphtheria, when there are just a few cases occurring in Bristol schools, think what it means to get into touch with their doctors, and either through them or, in unattended cases, directly by visits from the Medical Officer of Health, to have every single individual that has run the risk of being infected examined by the taking of cultures, and every infected person segregated. But how infinitely this work is multiplied when, owing to an outbreak developing before it is possible to limit it to the first victims, cases running into hundreds call for these exacting methods of control. Nevertheless, but few serious outbreaks have escaped being quickly brought under control, and our city thereby saved from the otherwise inevitable spread of this disease.

When we consider the vital importance of adequate research into the epidemiology, natural history and causation of these diseases, and the necessity for the closest co-ordination between the pathological laboratory and the Health Department, it is a matter for regret that, so far as we can gather from the Reports, facilities for pathological research are only available on special occasions, as an extra and occasional resort, rather than as an integral part of public health administration.

Bristolians should know, too, that as regards diphtheria theirs was the first city in England to undertake the routine bacteriological control of "contacts" and of infected cases; and that it was Dr. Heaven, assisting our M.O.H., who first drew attention to the importance of nasal diphtheria carriers as a possible source of epidemic outbreaks of diphtheria; and again, coming down to the present day, Dr. Davies was among the first in England to apply the Schick test for protecting his hospital staff against the dangers of diphtheritic infection.

We have but touched on the multifarious aspects of the work of the M.O.H.; obviously many of the newer developments of the department are largely due to his application of the latest discoveries in medical work.

Is it a matter for wonder that our old city's reputation stands so high in preventive medicine, seeing how greatly her Medical Officer has contributed to its evolution?

To those of us whose professional work affords the opportunity of watching the ever-increasing and widening of the Medical Officer of Health's responsibilities it is abundantly evident that the time has come when we cannot afford to let this important work outstrip the assistance afforded by the "City Fathers" for its maintenance and proper development.

THE EDITOR.

